# Medical Therapy in Patients with Heart Failure: A Delphi Consensus from Italian Cardiologists

**DOI:** 10.3390/jcm14165729

**Published:** 2025-08-13

**Authors:** Valentina Tardivo, Emanuele Venturini, Gaetano M. Ruocco, Guido Pastorini, Elisa Bertone, Mauro Feola

**Affiliations:** 1 School of Geriatry, University of Medicine Turin, 10126 Turin, Italy; valentinatardivo94@gmail.com; 2 School of Sport Medicine, University of Medicine Perugia, 06100 Perugia, Italy; emanuele.venturini92@gmail.com; 3 Cardiology Department I, Veris Delli Ponti Hospital, 73020 Scorrano, Italy; gmruocco@virgilio.it; 4 Department of Cardiology, Ospedale Regina Montis Regalis, Strada del Rocchetto 99, 12084 Mondovì, Italy; guido.pastorini@aslcn1.it (G.P.); elisa.bertone@aslcn1.it (E.B.)

**Keywords:** heart failure, medical therapy, Delphi method

## Abstract

**Background**: Adherence to current clinical guidelines is crucial for ensuring optimal therapy in patients with heart failure (HF). This study aims to explore how cardiologists, as specialists in heart failure, approach the clinical scenarios encountered in the management of HF patients, in line with the recommended guidelines. A heart failure-focused meeting was organized, during which participating cardiologists engaged actively. During HF meetings in which cardiologists participated, 108 questionnaires were distributed electronically. In total, 57 men and 51 women expressed their opinions regarding the Delphi analysis. **Results**: A strong consensus on the benefits of beta-blockers in improving prognoses for, and reducing mortality in, patients with HF and reduced systolic function emerged. The majority of cardiologists continue to prefer intravenous therapy with continuous loop-diuretic administration in combination with thiazide diuretics. The use of metolazone elicits fewer preferences, probably due to concerns about side effects. Certainly, SGLT2i is useful in reducing hospitalizations and reducing congestion; however, there is no full consensus on whether MRAi should be discontinued in favor of SGLT2i alone. The majority of participants would discontinue MRAs in the presence of hyperkalemia and worsening renal function, maintaining sacubitril/valsartan, and indicating a priority for renal safety. There was near-unanimous agreement on the early initiation of sacubitril/valsartan after the stabilization of patients hospitalized for heart failure. **Conclusions:** A significant majority (97%) of cardiologists expressed a preference for utilizing all of the guideline-recommended drug classes in the management of heart failure, even if this meant not always reaching the maximum tolerated dose for each medication. This approach underscores the importance of comprehensive therapy, targeting multiple pathophysiological mechanisms in heart failure. Cardiologists emphasized that while achieving optimal dosing is ideal, flexibility in treatment regimens is often necessary to accommodate individual patient characteristics, tolerance, and clinical status. The findings highlight the need for personalized treatment strategies that align with current guidelines, while also recognizing the challenges and variability in patient responses to therapy.

## 1. Introduction

Heart failure represents one of the major public health problems; it affects millions of people and imposes a significant economic and social burden [1]. In Italy, the prevalence of heart failure ranges from 2% to 3% of the general population [2]. However, this percentage increases significantly among individuals over the age of 65, reaching approximately 10–15%. There are about 1.5 million people affected by heart failure in our country and around 6 million people with heart failure in Europe.

The prevalence of heart failure is a significant issue both in Italy and across Europe, with trends reflecting the aging population and the increase in cardiovascular risk factors [3]. All of this underscores the importance of prevention strategies, early diagnosis, and correct management of the disease. The adherence to the European Society of Cardiology guidelines published in 2023 [4], which recommend a strategy including ‘four pillars,’ seems to be far from being achieved in the majority of our patients [5]. This article aims to describe how cardiologists, as experts in heart failure, can approach the clinical scenarios that may arise in the treatment of patients with heart failure using the recommended guidelines.

Despite the existence of well-established clinical guidelines for heart failure management, adherence in real-world practice remains variable. Reasons for this variability include differing institutional policies, clinician preferences, and unclear pathways of decision-making in clinical settings. Understanding who drives therapeutic choices and how clinical pathways are established is essential to improving implementation. The Delphi method offers a systematic approach to gather expert consensus where empirical data are limited, allowing for the identification of barriers to, and facilitators for, guideline adherence.

## 2. Study Design

The Delphi method has been extensively utilized to achieve consensus among experts, particularly in areas such as heart failure (HF) treatment where evidence may be incomplete or clinical practice heterogeneous [5,6]. This structured approach facilitates the integration of expert opinion to guide decision-making in complex clinical scenarios.

The present study aims to elucidate how cardiologists who specialize in heart failure manage various clinical situations in accordance with current guideline recommendations. The expert panel comprised 108 cardiologists from diverse regions of Italy, chosen randomly, who were all recognized for their expertise in HF management. The panel consisted of both general cardiologists and HF subspecialists. These experts practiced in academic medical centers (60%) and community hospitals (40%), ensuring a diverse and representative sample of real-world practice.

Experts were invited to respond anonymously to a series of statements via an electronically distributed questionnaire. The study was conducted in two phases: initially, a panel of HF experts (March 2023), on a clinical practice basis, developed statements covering four therapeutic domains: beta-blockers (3 statements), diuretics (3 statements), mineralocorticoid receptor antagonists (MRA) or sacubitril-valsartan (ARNI) (7 statements), and sodium-glucose cotransporter-2 inhibitors (SGLT2i) (2 statements). Respondents rated their level of agreement on a five-point Likert scale (1 = strongly disagree, 2 = disagree, 3 = somewhat agree, 4 = agree, and 5 = strongly agree). In the second phase, questionnaires were electronically distributed during HF meetings with active cardiologist participation. Responses were analyzed post-meeting. A predefined consensus threshold of ≥75% agreement—defined as the proportion of experts selecting “agree” or “strongly agree” (scores 4 or 5)—was used to identify statements with consensus. Statements not meeting this threshold were further reviewed and potentially revised.

## 3. Results

**Figure 1:**
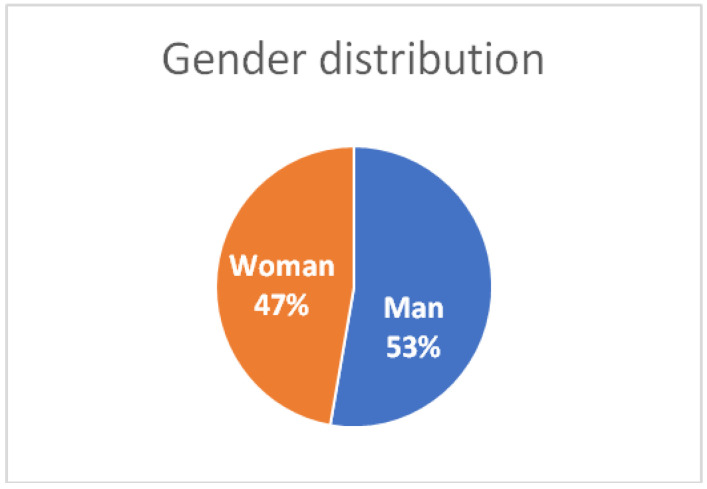
Gender distribution of responders. Interpretation: The near-equal gender distribution ensures balanced perspectives in the Delphi analysis.

A total of 108 cardiologists participated in the survey, with a gender distribution of 57 men and 51 women (Figure 1).

**Figure 2:**
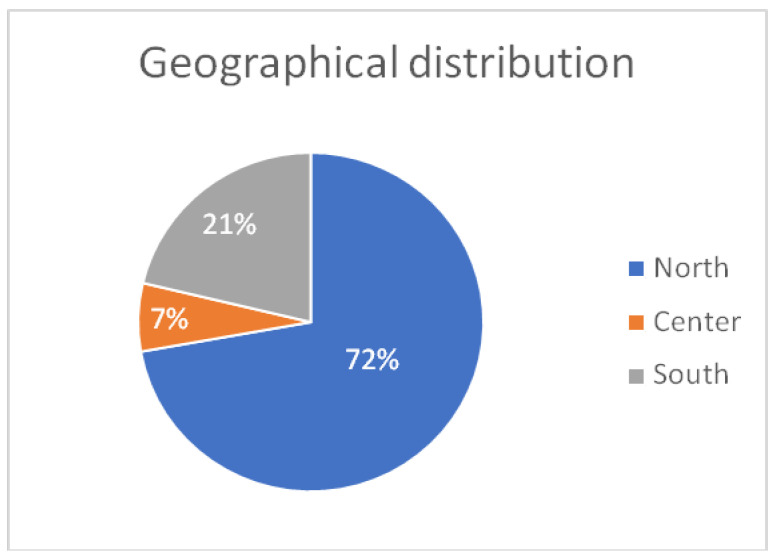
Geographical origin of responders. Interpretation: A predominance of participants from northern Italy may influence treatment preferences and highlights the need to consider regional variations in clinical practice.

Geographically, the majority (72, 66.6%) of experts came from northern Italy, while 7 (6.4%) were from central Italy and 23 (21.3%) from southern Italy (Figure 2).

This distribution reflects current regional differences in HF care infrastructure, with a predominance of centers and HF programs located in northern Italy. Although geographical trends in therapeutic responses were not statistically analyzed in this study, the regional imbalance may influence interpretation and deserves consideration in future analyses.

Regarding workplace setting, 98 cardiologists (90.1%) were employed in public hospitals, while 3 (2.8%) worked in private institutions with cardiology departments, and 5 (4.6%) were active in outpatient clinics.

**Figure 3:**
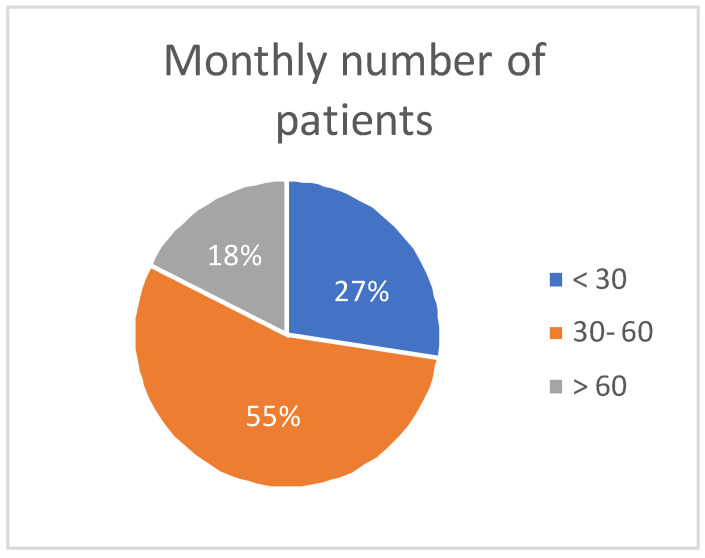
Monthly numbers of patients visited by responders. Interpretation: High patient volumes among respondents suggest a robust clinical experience, potentially affecting therapeutic decisions.

Clinical experience was indirectly assessed through patient volume: more than 50% of respondents reported managing at least 30 to 60 or more HF patients per month (Figure 3).

This suggests substantial hands-on experience with HF patients, which likely contributes to informed clinical decision-making. Although the impact of patient volume on therapeutic responses was not explored in this Delphi round, it represents a valuable direction for future subgroup analysis.

## 4. Discussion

Regarding statements in Table 1, which addresses beta-blocker therapy, the experts reached strong consensus on the significant benefits of beta-blockers in improving prognosis and reducing mortality in heart failure (HF) patients with reduced systolic function, regardless of gender (98% agreement), and even in the presence of atrial fibrillation (91%) [4]. Conversely, carvedilol was preferred in patients with HF and concomitant high blood pressure (79%) [7]. While beta-blockers are widely regarded as one of the fundamental pillars of HF therapy, there are documented differences in efficacy between men and women. In the context of ischemic cardiomyopathy, the beneficial effects of beta-blockers may be less pronounced in women compared to men. A potential factor contributing to the increased risk of HF among women may involve an interaction between hormone-replacement therapy and beta-blocker action. Specifically, progestin may inhibit the cardiac expression of β-1-adrenoceptors and diminish β-adrenergic-mediated stimulation, which could reduce cardiac output and predispose women to heart failure during acute coronary syndromes. Additionally, women may exhibit lower tolerance to beta-blocker therapy due to pharmacokinetic differences, such as enhanced drug absorption and a lower distribution volume, which are related to body composition and size differences. Furthermore, women tend to have higher resting levels of aortic wave reflection compared to men. Since beta-blockers increase wave reflections, this mechanism may potentially be harmful in women, particularly during acute ischemic events, by increasing left ventricular workload [4].

This Delphi-based analysis reveals a high degree of consensus among Italian cardiologists on several key aspects of heart failure (HF) management, particularly regarding the use of beta-blockers and SGLT2 inhibitors. Nevertheless, responses also highlight variability in certain clinical choices, suggesting the persistence of hesitancy in guideline implementation.

As shown in Table 2, a strong consensus (88%) was reached on the statement that beta-blocker therapy improves prognosis regardless of gender, with a median agreement score of 5. However, the statement regarding beta-blocker effectiveness in reducing mortality for patients with atrial fibrillation showed only partial consensus (72%), reflecting some variability in expert opinion. Notably, no consensus was achieved for the preference of carvedilol in patients with high blood pressure, which may indicate diverse prescribing habits or uncertainties in this clinical scenario.

Concerning statements in Table 3 on the use of diuretics, the majority of cardiologists (83%) favored intravenous therapy with continuous loop diuretic infusion. This strong preference is consistent with the existing literature, particularly in acute clinical settings, where effective and rapid control of fluid overload is critical [8]. However, the preference for continuous infusion has not been supported by findings from the DOSE trial, which did not show any significant difference in efficacy between continuous infusion and bolus administration. Moreover, continuous infusion was not associated with improvement in secondary outcomes, such as net diuresis, weight loss, or treatment failure. In contrast, high-intensity furosemide therapy (2.5 times the oral dose) was associated with substantial improvements in net diuresis, weight loss, and symptom relief, as compared to low-intensity treatment [8]. While continuous intravenous infusion of loop diuretics is favored (83%), this contrasts with findings from the DOSE trial, which demonstrated no superiority over bolus administration [8]. The continued preference for continuous infusion may reflect a disconnect between clinical experience and randomized trial data, or a perceived better tolerance profile in specific patient populations.

Furthermore, a large majority (82%) of respondents expressed a preference for the use of loop diuretics in combination with thiazide diuretics. This combination strategy appears to be effective in overcoming diuretic resistance, a common complication in patients with refractory edema, due to its synergistic effects on sodium reabsorption at different nephron sites [9]. However, the use of metolazone showed less preference compared to the other two diuretic strategies, suggesting that concerns about its potential side effects influenced the respondents’ choice. This illustrates how perceived risk and lack of real-time safety monitoring tools may impede broader adoption of guideline-suggested therapies. As demonstrated in the literature, clinical experiences with adding metolazone during acute decompensated heart failure (ADHF) treatment have been associated with increased collateral effects and, independently, with increased mortality. This clinical disadvantage is primarily attributed to the induction of hyponatremia, hypokalemia, and worsening renal function. These findings suggest that combining diuretic therapy with metolazone carries inherent risks and may not be suitable for all patients [10]. A recent retrospective study by Palazzuoli et al. highlighted that patients treated with metolazone required intravenous saline supplementation due to electrolyte imbalances that emerged during treatment [11].

Table 4 presents the level of consensus regarding in-hospital diuretic use among the panelists. Notably, none of the statements reached the predefined consensus threshold of 75%. For instance, only 49% agreed on the preference for continuous intravenous loop diuretics due to fewer side effects, while 57% favored combining IV loop diuretics with thiazides to enhance efficacy. The lowest agreement (30%) was observed for the cautious use of metolazone due to safety concerns. These findings suggest variability and uncertainty among Italian cardiologists in the optimal diuretic management during hospitalization for heart failure, reflecting an area where further evidence and guidance may be needed.

Table 5 summarizes expert opinions on the use of sodium-glucose cotransporter-2 inhibitors (SGLT2i) in heart failure management. The panel did not reach consensus on initiating SGLT2i over mineralocorticoid receptor antagonists (MRAs) in patients with advanced heart failure and chronic congestion, with only 38% agreement. However, a higher agreement (86%) was observed for the strategy of reducing loop diuretic dosage when prescribing SGLT2i. These results indicate cautious adoption of SGLT2i in clinical practice, highlighting areas for further clinical clarification and education.

Concerning statements in Table 6, respondents indicated a general reluctance to initiate mineralocorticoid receptor antagonist (MRA) therapy before, or in place of, SGLT2 inhibitor monotherapy in patients with chronic congestion. These discrepancies highlight the need for education and institutional support to reconcile evidence-based recommendations with bedside practice. Cumulative percentages reveal that a significant portion (28%) placed their responses in the lower categories (1 and 2), while 62% ranked their responses in the higher categories (3 to 5). The DAPA-HF [12], EMPEROR-Reduced [13], and EMPEROR-Preserved [14] trials have demonstrated significant clinical benefits with the addition of SGLT2 inhibitors to heart failure therapy, with no evidence supporting the discontinuation of MRAs in these patients. In recent years, Packer has criticized the conventional sequential approach to implementing these therapies, arguing that it is historically driven and not sufficiently evidence-based [15]. He proposed an accelerated, evidence-based three-step approach: the simultaneous initiation of a beta-blocker and SGLT2 inhibitor, followed by sacubitril/valsartan in 1–2 weeks, and then the introduction of an MRA 1–2 weeks later. These last two steps can be reordered or compressed based on individual patient circumstances. Rapid sequencing of these therapies is a novel strategy that could dramatically improve the timely implementation of treatments that reduce morbidity and mortality in patients with heart failure with reduced ejection fraction [15].

Interestingly, the responders agree with the necessity to reduce loop-diuretic therapy after starting a SGLT2i administration. This opinion is widely shared among cardiologists. In many patients, the additional administration of an SGLT2 inhibitor to HF patients allowed a reduction in the dosage of diuretics (Table 6).

Conversely, a significant proportion (56%) of responders to statements in Table 6 did not lean towards reducing the loop diuretic dosage when prescribing MRAs. This suggests a lack of overwhelming consensus on adjusting loop diuretics in this situation. Although loop diuretics remain the diuretic of choice for the treatment of patients with heart failure, the reduced response or resistance to these is an underestimated issue, which can lead to a worse clinical course and prolonged hospital stay. This common clinical scenario requires an increased dose of diuretic for decongestion. Although loop diuretics have not demonstrated a mortality benefit in HF [16], Testani et al., who highlighted the prognostic meaning of diuretic response efficiency in acute decompensated HF, have shown that the evidence of resistance to diuretics results in a poor prognosis with higher expected mortality and an increased risk of rehospitalization for HF [17]. Resistance to diuretics and reduced response to loop diuretics are, unfortunately, not uncommon in clinical practice. In fact, guidelines do not specify which type of loop diuretic infusion should be used, and we contend that it is desirable to use the lowest possible dose to achieve the desired effect.

Mineralocorticoid receptor antagonists (MRAs), such as spironolactone and eplerenone, are common drugs used in chronic HF to reduce adverse clinical outcomes. However, the doses commonly used have minimal diuretic effect, with their prognostic benefits likely resulting from their neurohormonal antagonism. In conclusion, loop diuretics remain the cornerstone of diuretic therapy. Under specific conditions of treatment resistance, the introduction of thiazide and MRAi diuretics can be evaluated to allow an effective “sequential blockade” of the nephron [18].

In fact, a substantial majority (72%) would discontinue MRAs in the presence of hyperkalemia and worsening renal function, while maintaining sacubitril/valsartan (Table 7). This suggests a clear preference for prioritizing kidney safety in this scenario. Heart failure itself is associated with a high risk of renal dysfunction and the development of CKD. Conversely, poor renal function has been shown to predict left ventricular (LV) dysfunction. Collectively, the bidirectional link between cardiac and renal function can lead to clinical presentations that are termed cardio-renal syndrome (CRS). Hyperkaliemia increases the risk of arrhythmias, morbidity, and mortality. Concurrent use of aldosterone antagonists with ACEi increases the risk of hyperkaliemia; thus, they should be used with caution [19].

A large majority (87%) of responders used to initiate Patiromer or Sodium zirconium cyclosilicate to manage hyperkalemia while continuing other heart failure therapies. Several studies have shown the positive effect and safety of Patiromer or Sodium zirconium cyclosilicate in managing hyperkalemia [20]. Both drugs showed a rapid onset of action. Regarding safety, the most common adverse events associated with these drugs were gastrointestinal in nature, such as constipation (most frequently reported with Patiromer) and edema. However, overall, the drugs were considered generally well tolerated in the studies analyzed [20].

An overwhelming majority (94% of respondents) agreed with initiating sacubitril/valsartan promptly after stabilizing hospitalized patients with heart failure. This reflects strong support for the early use of this therapy in the inpatient setting. The TRANSITION study [21] examined the safety and tolerability of the early initiation of sacubitril/valsartan therapy in heart failure patients with reduced ejection fraction (HFrEF) who were hemodynamically stable and hospitalized for an episode of acute heart failure (ADHF), or who received the drug shortly after discharge. The study showed that early initiation of sacubitril/valsartan in patients with HFrEF hospitalized for ADHF was safe and well tolerated. A significantly greater proportion of patients in the early-onset group achieved the target dose of 200 mg twice daily at 10 weeks, compared to the late-onset group. The TRANSITION study supports early initiation of sacubitril/valsartan in patients with hemodynamically stable HFrEF during hospitalization for ADHF.

An even larger majority (97% of responders) supported an early titration of sacubitril/valsartan in patients with HF with systolic dysfunction, even suggesting telemedicine (Table 8). The main obstacles include hypotension/side effects, physician inertia, treatment complexity, and individual patient characteristics. Education of patients, adaptation of structured titration protocols, implementation of close monitoring whenever possible, and, finally, a multidisciplinary approach to optimize titration seemed to be the correct hypothesis for obtaining a complete compliance with the treatment. This highlights the importance of the up-titration of the therapy in those patients. The recommended doses of key drugs (ACE inhibitors/ARBs/ARNIs, beta-blockers, MRAs) for HFrEF are based on studies demonstrating reduced mortality and morbidity. In clinical practice, many patients with HFrEF do not achieve the recommended target doses. This phenomenon, documented by Savarese et al., is attributed to factors such as delayed initiation of therapy, insufficient titration, and treatment discontinuity, often due to concerns about side effects and the lack of timely decision-making support [22]. The main obstacles include hypotension/side effects, physician inertia, treatment complexity, and individual patient characteristics. Education of patients, adaptation of structured titration protocols, implementation of close monitoring whenever possible, and, finally, a multidisciplinary approach to optimize titration seemed to be the correct hypothesis for obtaining a complete compliance with the treatment.

Finally, 97% of cardiologists expressed a preference for using all the guideline-recommended drug classes, even if they did not reach the maximum tolerated dose. This reflects a strategy of using a combination approach to achieve benefits from multiple mechanisms of action.

## 5. Conclusions

In conclusion, this Delphi analysis emphasized the utilization of guideline-directed medical therapy (GDMT) for HF, including sacubitril/valsartan, and even in different scenarios. Strategies to manage hyperkalemia that enable the continued use of beneficial therapies are favored. While aiming for optimal dosing is desirable, using a combination of drug classes is often prioritized, even if it means using less than the maximum dose of each.

Furthermore, the analysis highlighted a strong consensus on the benefits of beta-blockers in improving prognosis and reducing mortality in patients with HF and reduced stroke function, regardless of the presence of atrial fibrillation. In particular, carvedilol seemed to be preferred in patients with HF and hypertension.

The majority of cardiologists, in spite of the results of DOSE Trial, continue to prefer the therapy with intravenous continuous loop-diuretics administration and the combination with thiazide diuretics.

Specifically, the use of metolazone elicits fewer preferences, probably due to concerns about side effects.

Undeniably, SGLT2i are helpful in reducing hospitalizations and congestion. Yet, experts could not find a total consensus regarding a possible abandonment of the use of MRAi diuretics in favor of SGLT2i alone.

The majority of participants would discontinue MRAs in the presence of hyperkalemia and worsening renal function, maintaining sacubitril/valsartan, and indicating a priority for renal safety. The addition of Patiromer or sodium and zirconium cyclosilicate to manage hyperkalemia seemed to be the better choice in order to continue other therapies for heart failure.

Ultimately there is almost unanimous consensus on the early initiation of sacubitril/valsartan after the stabilization of patients hospitalized for heart failure, its early titration (including via telemedicine), and its continuation in patients even in case of improvement in cardiac function.

These findings suggest a need for targeted educational initiatives to address persistent hesitancy in implementing guideline-directed therapies, focusing on concerns about side effects and treatment optimization.

Moreover, the observed preference patterns, such as greater SGLT2i uptake in some settings, may reflect the influence of institutional policies, highlighting the importance of organizational factors in therapeutic decision-making.

Future directions could include integrating this Delphi framework into a national registry to monitor evolving clinical practices, with annual repetitions to assess changes over time and the impact of educational or policy interventions.

## Figures and Tables

**Table 1:** Use of Beta-blockers.

	1	2	3	4	5	TOT
In patients with HFrEF, beta-blocker therapy improves the prognosis regardless of gender	0	1	11	27	60	99
	2%		98%			100%
In patients with heart failure with reduced systolic function, beta-blocker therapy reduces mortality in those with sinus rhythm as well as in those with atrial fibrillation	1	7	19	29	42	98
	9%		91%			100%
I prefer to use carvedilol in patients with heart failure (with reduced or preserved systolic function) if they present high blood pressure	5	15	35	24	18	97
	21%		79%			100%

**Table 2:** Consensus Levels and Quartile Distribution for Clinical Statements on Heart Failure Therapy.

Statement	% Consensus (4 + 5)	Q1	Median (Q2)	Q3	Notes
1. Beta-blocker improves prognosis regardless of gender	88%	3	5	5	Consensus reached
2. Beta-blocker reduces mortality in sinus rhythm and AF	72%	3	4	5	Partial consent
3. Prefer carvedilol if high BP in HF patients	43%	2	3	4	Consensus not reached

**Table 3:** Use of diuretics.

	1	2	3	4	5	TOT
In hospitalized patients with heart failure and signs of congestion, I prefer an intravenous therapy with loop diuretics in continuous infusion as I believed it is associated with fewer side effects	5	11	34	36	11	97
	17%		83%			100%
In hospitalized patients with heart failure and signs of congestion, I prefer intravenous therapy with loop diuretics in combination with thiazide diuretics in order to obtain a diuretic effect at multiple sites of the nephron, thus improving the efficacy of diuretic therapy	3	10	28	33	21	95
	18%		82%			100%
In hospitalized patients with heart failure and signs of congestion I prefer to use less metolazone because I am concerned about increased side effects such as hyponatremia, hypokalemia, and acute kidney failure	7	29	29	18	10	93
	39%		61%			100%

**Table 4:** Consensus on In-Hospital Diuretic Use.

Statement	% Agreement (4 + 5)	Q1	Median (Q2)	Q3	Notes
1. Prefer continuous IV loop diuretics for fewer side effects	49%	3	3	4	Consensus not reached
2. Prefer IV loop + thiazides to enhance efficacy	57%	3	4	5	Consensus not reached
3. Use less metolazone due to safety concerns	30%	2	3	4	Consensus not reached

**Table 5:** Use of SGLT2i.

	1	2	3	4	5	TOT
In patients with advanced heart failure and signs of chronic congestion, I prefer to start with a SGLT2 inhibitor rather than a mineralocorticoid receptor antagonist in order to preserve hemodynamics	17	18	32	18	9	94
	38%		62%			100%
In patients with heart failure, when I prescribe SGLT2 inhibitors, I always reduce the dosage of loop- diuretics	3	10	22	31	27	93
	14%		86%			100%

SGLT2i: Sodium Glucose Cotransporter 2 Inhibitors.

**Table 6:** Use of SGLT2 inhibitors (SGLT2i).

Statement	% Agreement (4 + 5)	Q1	Median (Q2)	Q3	Notes
1. Prefer SGLT2i over MRA in chronic congestion to preserve hemodynamic	29%	2	3	4	Consensus not reached
2. Always reduce loop diuretics when prescribing SGLT2i	62%	3	4	5	Consensus not reached

**Table 7:** Use of MRA and ARNi.

	1	2	3	4	5	TOT
In patients with heart failure, when I prescribe MRAs, I always reduce the dosage of the loop diuretic	21	31	31	9	2	94
	56%		44%			100%
In patients with heart failure and reduced systolic function, in case of developing hyperkalemia with associated worsening of renal function, I discontinue MRAs while maintaining therapy with sacubitril/valsartan	11	15	33	28	6	93
	28%		72%			100%
In patients with reduced systolic cardiac function, if hyperkalemia has occurred, I prefer to use “Patiromer” or “Sodium zirconium cyclosilicate” while maintaining therapy with ACE/RAASi/ARNI and MRA	5	7	19	23	39	93
	13%		87%			100%
In hospitalized patients with heart failure, after adequate hemodynamic stabilization, therapy with sacubitril/valsartan should be initiated immediately	1	4	7	31	50	93
	6%		94%			100%
In patients with chronic heart failure and reduced diastolic function, the drug dose should be titrated as early as two weeks after starting therapy with sacubitril/valsartan, possibly using even using telemedicine.	2	0	7	37	47	93
	3%		97%			100%
In patients with chronic heart failure with reduced systolic function who have developed reverse remodeling and, therefore, have shown total or partial recovery of systolic function, therapy with sacubitril/valsartan is always continued.	1	0	3	18	70	92
	2%		98%			100%
In patients with heart failure, I prefer to use all pharmacological classes (BB/ACEi/RAASi/ARNI, MRA, SGLT2) even if I do not obtain the maximum therapeutic dose for each of them	1	1	7	17	67	93
	3%		97%			100%

MRA: Mineralocorticoid Receptor Antagonist; ARNI: Angiotensin Receptor Neprilysin Inhibitor.

**Table 8:** Expert Agreement and Response Distribution on MRA and ARNI Therapy.

Statement	% Agreement (4 + 5)	Q1	Median (Q2)	Q3	Notes
1. Always reduce loop diuretics when prescribing MRAs	12%	2	3	3	Consensus not reached
2. Discontinue MRAs in case of hyperkalaemia + renal worsening, maintain sac/val	37%	2	3	4	Consensus not reached
3. Use potassium binders (e.g., Patiromer) to maintain full HF therapy	67%	3	4	5	Consensus not reached
4. Initiate sac/val immediately after hemodynamic stabilization	87%	4	5	5	Consensus reached
5. Titrate sac/val after 2 weeks, possibly via telemedicine	90%	4	5	5	Consensus reached
6. Continue sac/val in patients with reverse remodelling	96%	5	5	5	Consensus reached
7. Prefer all drug classes even without max dose	91%	4	5	5	Consensus reached

## Data Availability

Date are available in database of G.M.R.

## References

[1] Savarese G., Becher P.M., Lund L.H., Seferovic P., Rosano G.M.C., Coats A.J.S. (2023). Global burden of heart failure: A comprehensive and updated review of epidemiology. Cardiovasc. Res..

[2] Saglietto A., Manfredi R., Elia E., D’Ascenzo F., De Ferrari G.M., Biondi-Zoccai G., Munzel T. (2021). Cardiovascular disease burden: Italian and global perspectives. Minerva Cardiol. Angiol..

[3] Piccinni C., Antonazzo I.C., Simonetti M., Mennuni M.G., Parretti D., Cricelli C., Colombo D., Nica M., Cricelli I., Lapi F. (2017). The Burden of Chronic Heart Failure in Primary Care in Italy. High. Blood Press. Cardiovasc. Prev..

[4] Bugiardini R., Yoon J., Kedev S., Stankovic G., Vasiljevic Z., Miličić D., Manfrini O., van der Schaar M., Gale C.P., Badimon L. (2020). Prior Beta-Blocker Therapy for Hypertension and Sex-Based Differences in Heart Failure Among Patients with Incident Coronary Heart Disease. Hypertension.

[5] Hasson F., Keeney S., McKenna H. (2000). Research guidelines for the Delphi survey technique. J. Adv. Nurs..

[6] Nair R., Aggarwal R., Khanna D. (2011). Methods of formal consensus in classification/diagnostic criteria and guideline development. Semin. Arthritis Rheum..

[7] Belfort D.S.P., Bocchi E.A., Cafezeiro C.R.F., Wanderley M.R.B., Salemi V.M.C., Rocon C., Munhoz R.T., Chizzola P.R., Biselli B., Furlan D.A.G. (2025). Carvedilol as Single Therapy for Heart Failure with Improved Ejection Fraction: A Randomized Clinical Trial (CATHEDRAL-HF). JACC Heart Fail..

[8] Felker G.M., Lee K.L., Bull D.A., Redfield M.M., Stevenson L.W., Goldsmith S.R., LeWinter M.M., Deswal A., Rouleau J.L., Ofili E.O. (2011). Diuretic Strategies in Patients with Acute Decompensated Heart Failure. N. Engl. J. Med..

[9] Brater D.C. (1998). Diuretic therapy. N. Engl. J. Med..

[10] Brisco-Bacik M.A., ter Maaten J.M., Vedage N.A., Wilson F.P., Testani J.M. (2017). The Increased Mortality Risk Associated with Metolazone in Acute Heart Failure is mediated by Worsening Renal Function and Electrolyte Disturbances. J. Card. Fail..

[11] Palazzuoli A., Ruocco G., Severino P., Gennari L., Pirrotta F., Stefanini A., Tramonte F., Feola M., Mancone M., Fedele F. (2021). Effects of Metolazone Administration on Congestion, Diuretic Response and Renal Function in Patients with Advanced Heart Failure. J. Clin. Med..

[12] McMurray J.J.V., Solomon S.D., Inzucchi S.E., Køber L., Kosiborod M.N., Martinez F.A., Ponikowski P., Sabatine M.S., Anand I.S., Bělohlávek J. (2019). Dapagliflozin in Patients with Heart Failure and Reduced Ejection Fraction. N. Engl. J. Med..

[13] Packer M., Anker S.D., Butler J., Filippatos G., Pocock S.J., Carson P., Januzzi J., Verma S., Tsutsui H., Brueckmann M. (2020). Cardiovascular and Renal Outcomes with Empagliflozin in Heart Failure. N. Engl. J. Med..

[14] Anker S.D., Butler J., Filippatos G., Ferreira J.P., Bocchi E., Böhm M., Brunner-La Rocca H.P., Choi D.J., Chopra V., Chuquiure-Valenzuela E. (2021). Empagliflozin in Heart Failure with a Preserved Ejection Fraction. N. Engl. J. Med..

[15] Packer M., McMurray J.J.V. (2021). Rapid evidence-based sequencing of foundational drugs for heart failure and a reduced ejection fraction. Eur. J. Heart Fail..

[16] Chiong J.R., Cheung R.J. (2010). Loop diuretic therapy in heart failure: The need for solid evidence on a fluid issue. Clin. Cardiol..

[17] Testani J.M., Brisco M.A., Turner J.M., Spatz E.S., Bellumkonda L., Parikh C.R., Tang W.H. (2014). Loop diuretic efficiency: A metric of diuretic responsiveness with prognostic importance in acute decompensated heart failure. Circ. Heart Fail..

[18] Pham D., Grodin J.L. (2017). Dilemmas in the Dosing of Heart Failure Drugs: Titrating Diuretics in Chronic Heart Failure. Card. Fail. Rev..

[19] Al-Naher A., Wright D., Devonald M.A.J., Pirmohamed M. (2018). Renal function monitoring in heart failure—What is the optimal frequency? A narrative review. Br. J. Clin. Pharmacol..

[20] Meaney C.J., Beccari M.V., Yang Y., Zhao J. (2017). Systematic Review and Meta-Analysis of Patiromer and Sodium Zirconium Cyclosilicate: A New Armamentarium for the Treatment of Hyperkalemia. Pharmacotherapy.

[21] Wachter R., Senni M., Belohlavek J., Straburzynska-Migaj E., Witte K.K., Kobalava Z., Fonseca C., Goncalvesova E., Cavusoglu Y., Fernandez A. (2019). Initiation of sacubitril/valsartan in haemodynamically stabilised heart failure patients in hospital or early after discharge: Primary results of the randomised TRANSITION study. Eur. J. Heart Fail..

[22] Savarese G., Lindberg F., Cannata A., Chioncel O., Stolfo D., Musella F., Tomasoni D., Abdelhamid M., Banerjee D., Bayes-Genis A. (2024). How to tackle therapeutic inertia in heart failure with reduced ejection fraction. A scientific statement of the Heart Failure Association of the ESC. Eur. J. Heart Fail..

